# Tick-Bite “Meteo”-Prevention: An Evaluation of Public Responsiveness to Tick Activity Forecasts Available Online

**DOI:** 10.3390/life13091908

**Published:** 2023-09-14

**Authors:** Petr Zeman

**Affiliations:** Medical Laboratories, Konevova 205, 130 00 Prague, Czech Republic; zeman3@post.cz

**Keywords:** tick-borne diseases, tick bite, *Ixodes ricinus*, prevention

## Abstract

Until causal prophylaxis is available, the avoidance of ticks and personal protection provide the best insurance against contracting a tick-borne disease (TBD). To support public precaution, tick-activity forecasts (TAFs) based on weather projection are provided online for some regions/countries. This study—aimed at evaluating the efficacy of this preventative strategy—was conducted between 2015 and 2019, and included two countries where TAFs are issued regularly (Czech Republic, Germany) and two neighbouring countries for reference (Austria, Switzerland). Google Trends (GT) data were used to trace public concern with TAFs and related health information. GTs were compared with epidemiological data on TBD cases and tick bites, wherever available. Computer simulations of presumable effectiveness under various scenarios were performed. This study showed that public access to TAFs/preventive information is infrequent and not optimally distributed over the season. Interest arises very early in midwinter and then starts to fall in spring/summer when human–tick contacts culminate. Consequently, a greater number of TBD cases are contracted beyond the period of maximum public responsiveness to prevention guidance. Simulations, nevertheless, indicate that there is a potential for doubling the prevention yield if risk assessment, in addition to tick activity, subsumes the population’s exposure, and a real-time surrogate is proposed.

## 1. Introduction

Hard ticks, particularly of the genus *Ixodes* (Acari: Ixodidae), endanger human health by sucking blood and transmitting a range of pathogens including the tick-borne encephalitis virus, spirochaetes of the *Borrelia burgdorferi* complex, rickettsial organisms such as *Anaplasma phagocytophilum* or *Rickettsia helvetica*, and other agents [1,2]. Moreover, they possess a unique repertoire of bioactive molecules in salivary secretions, making their feeding silent and the transfer of pathogens effectual [3]. Since a specific prophylaxis against almost all of these infections is unavailable to date, the personal protection and avoidance of host-seeking ticks remain to be essential for disease prevention [4]. One contemporary approach utilizes a well-known fact that host-seeking activity is weather-dependent [5,6], and employs short-term weather forecast data for predicting tick activity for the couple of days ahead; this cautionary information is disseminated to the general public via the Internet [7]. It is expected that risk-aware people will avoid contact with ticks and/or apply personal protective measures the more keenly the higher-tick-questing activity is anticipated—in other words—they will make the amount of exposure to the risk inversely proportional to the rate of the risk itself. This concept has been put into action in the prevention of *Ixodes ricinus* bites in two Central European countries, the Czech Republic and Germany, where it became a complement to routine weather forecasts [8,9].

So far, no study has evaluated the efficacy of this preventative strategy, partly because conventional methodologies would require an extensive, nation-wide questionnaire investigation. With the recent availability of Google Trends (GT) data, an alternative approach of inquiring into peoples’ health behaviour has become possible [10]. It takes advantage of the fact that people search daily for myriad themes on Google—a leading Internet search engine—seeking, among other things, for information that would help them safeguard their health. Every search that is undertaken, and every page that is viewed, is tracked. Google can retrospectively analyse a representative portion of these searches and provide data on geospatial and temporal patterns in search volumes for user-specified keywords [11]. Since the Internet activity mirrors people’s actual needs and interests, it is in this way possible to directly monitor particular aspects of population behaviour traced by appropriately chosen keywords, and to detect various patterns, trends, and connections to other (real-word) phenomena [12]. It was shown that there indeed exists a parallelism between trends detected in GT-data and trends revealed through conventional public surveys [13]. The evidential potential of GT-data has also been demonstrated in a variety of health-related applications [14].

GT is particularly suitable for investigating the performance of the tick-bite prevention system in question that is itself dependent on Internet searches. In the field of tick-borne diseases (TBDs), successful application of these types of search trend data have already been reported, though in different contexts. For example, clear seasonal patterns, correlated with those in reported disease cases, were detected in search volumes of the “Lyme disease/borreliosis” and “tick-borne encephalitis” keywords in the USA, Finland, and Germany, suggesting that the search dynamics could serve as a supplementary source of information for the disease surveillance [15,16,17,18,19]. As demonstrated for Germany, Sweden, and the USA, an increase in searching for “tick bite” or early disease symptoms on the Internet can alert the health system to a TBD’s upsurge a couple of weeks earlier than it is signalled through routine epidemiological monitoring, while also reflecting cases eluding registration [20,21,22,23]. Similarly, an increase in searching for “tick” and “mice” in Poland has been shown to presage an increase in the country’s level of morbidity of Lyme borreliosis in the same and following years, respectively [24]. On a European level, searches for national equivalents of the keyword “tick” were shown to approximately follow tick seasonal activity in local climatic conditions—subject to capricious usage of “tick” synonyms [25].

In this study, GT-data are used to assess search activities associated with the prevention of tick bites/TBDs in four countries of Central Europe—the two in which regular forecasts of risky weather have been published online, and another two where it is unavailable. Seasonal patterns in the searches and/or in epidemiological data are compared to each other, and computer simulation is performed to evaluate the potential of this prevention strategy to reduce the number of newly acquired disease cases. This study, however, does not address validation of the underlying weather and tick activity forecasts upon which this prevention measure depends.

## 2. Materials and Methods

### 2.1. Google Trends Data

GT retrieves data in the form of time series of indices (relative search volumes; RSVs) ranging between 0 and 100, with the highest search activity scored at 100 and all other search activities expressed relative to it. The data can be retrieved for any period from 2004 to the present (the longer time series, the coarser the temporal resolution) [11]. For the purpose of this study, the data were optimized to span over 5 years with weekly resolution, covering 2015–2019, a period when the Internet-based prevention systems in the Czech Republic and Germany had already been established, and before the public response could have been influenced by the COVID-19 pandemic. In addition, data on Austria and Switzerland—two neighbouring countries with similar TBDs’ epidemiology, but where this prevention system has not been implemented—were used for comparison. The keywords used to monitor population behaviour can be classified with respect to exposure to tick bites into 3 categories: (1) preventive searches, (2) post-exposure searches, and (3) searches associated with recreational/outdoor activity (Table 1).

### 2.2. Epidemiological Data

Official data on the incidence of TBDs in the Czech Republic, Germany, and Switzerland were obtained from national authorities [26,27,28]. Austrian data were not available at a sufficiently detailed temporal resolution [29]. Tick-borne encephalitis (TBE) universally, and Lyme borreliosis (LB) in the Czech Republic and Germany (in 9 out of 16 federal states), are notifiable diseases. In contrast, Swiss data on LB originate from a sentinel system—a network of medical practitioners-volunteers, whose reports are filtered to represent primarily acute forms of the disease (ECM, lymphocytoma), thus giving a sharper picture of LB seasonality [30]. The Swiss sentinel surveillance system is also unique in that it simultaneously collects data on patients that sought consultation for tick bites, which facilitated calibration of the tick-bite-to-case report lag parameter.

### 2.3. Weather-Derived Tick-Activity Index (TAI)

A complete record of the Internet forecasts was not available. To restore the past series of tick-questing activity, TAIs were calculated retrospectively from archived meteorological data. According to the concept of Daniel et al., 2010 [7], the questing activity of *I. ricinus* is driven by a fixed phenological cycle (Pc), and it boosted or hindered by instantaneous weather conditions, formally TAI = Pc(*t*) × *f*(**m**), where *t* denotes time of year and **m** is a vector of meteorological variables. In this study, Pc is a splined empirical distribution of *I. ricinus* activity averaged over a sample period (6 years), and f represents a modulating function—polynomial regression of tick activity (proportionated to Pc) against selected meteorological factors. Mean and minimum temperatures were extracted, and daily temperature range and antedescent precipitation index were calculated from daily gridded data archived by EU’s Climate Change Service (https://surfobs.climate.copernicus.eu/dataaccess/access_eobs.php (accessed on 20 July 2020)). Authentic tick data used to train the Czech tick-bite prevention system [31] were utilised for the model calibration, reaching a fit of 76.4%. Relying on geographical and ecological proximity [32], the same model was applied to estimate mean TAI for Bohemia as well as Bavaria—two bordering regions contributing the most to national TBDs’ morbidities. Areas situated >650 m a.s.l. were excluded from TAI estimations.

### 2.4. Statistical Analysis

GT-data present a peculiar data type requiring adequate approaches [33]. RSVs give no information on the population size (i.e., on the number of searches undertaken), only on proportions. In order to be able to rank the keywords according to the frequency of their usage, two indices were calculated: (1) the sum total of all RSVs over the study period, and (2) the proportion of weeks in which RSV > 0. Only data on sufficiently frequented keywords—with the latter index being greater than 20%—were further analysed. To be mutually comparable, the GT-, epidemiological-, and TAI data were aggregated over the 5 years study period, and the resulting average seasonal distributions standardized so that they all had an area under the curve (AUC) equal to unity. For the purpose of delimiting their culmination, a “summit period” (SP) is herein defined as an interval flanking 50% of the AUC’s highest segment (for details, see Supplementary File S1). A sliding window of fixed width of 5 weeks and two-sample Wilcoxon (signed rank) test were used for week-by-week comparison of the seasonal distributions. Briefly, for each week of the year w_i_, data sub-sets of size *n* = 25 (i.e., 5 consecutive weeks centred on w_i_ × 5 years of observation) were taken, tested for difference using the Wilcoxon test, and the window shifted a week ahead, treating the ends of the time series in a circular manner. Alternatively, for each seasonal distribution, an empirical cumulative distribution function with bootstrapped confidence limits (N_sim_ = 1000) was computed, and those whose confidence bands reflecting the 95% simultaneous confidence level did not overlap were considered divergent.

### 2.5. Computer Simulation

Computer models were used to simulate tick-bite- (or disease-) incidence under various preventative scenarios. All scenarios assumed that the tick-bite risk prediction is 100% correct, warning urgency is set proportional to risk rate, and that people behave accordingly.

#### 2.5.1. Model 1

The first model simulated the incidence, I, on the assumption that the risk rate is derived exclusively from tick activity (and translated straight to warning urgency). The activity of ticks is naturally changing in time and space—that is, high risk prevails in some situations, low risk in others—and the overall efficacy of the prevention system thus depends on the distribution of that risk integrated over all situations within a given area. This distribution was modelled with the beta distribution, B(.), in which parameters a and b were allowed to vary:

I = E(B(a, b) × (1 − B(a, b)))(1)

where the first right-hand term represents the variable risk rate and the second one represents the inversely proportional exposure to it. The efficiency was assessed from a comparison with the same model, assuming that people have no information on the risk and behave spontaneously/randomly:

I_0_ = E(B(a, b) × U()), (2)

where U() represents a uniform distribution.

#### 2.5.2. Model 2

The second model simulated the incidence on the assumption that both the tick and human activities carry a weight, and the level of warning urgency is derived from the human-tick encounter rate. In these simulations, empirical distributions of the tick-questing activity, Pc(.), and human outdoor activity, Ha(.), were utilised. To measure the effect of either factor on the prevention efficacy, they were combined using weighed geometric mean and re-normalised: P_H_ = (Pc(1 − W) × HaW)* (W is a weight ranging between 0 and 1, and “*” symbolizes normalisation). The incidence was then computed as

I = E(P_c_(X) × (1 − P_H_(X))), (3)

where X represents points in time randomly drawn from the distribution of Ha. All computations were conducted in R (https://www.r-project.org/ (accessed on 10 July 2023)).

## 3. Results

### 3.1. Data Analysis

**Figure 1:**
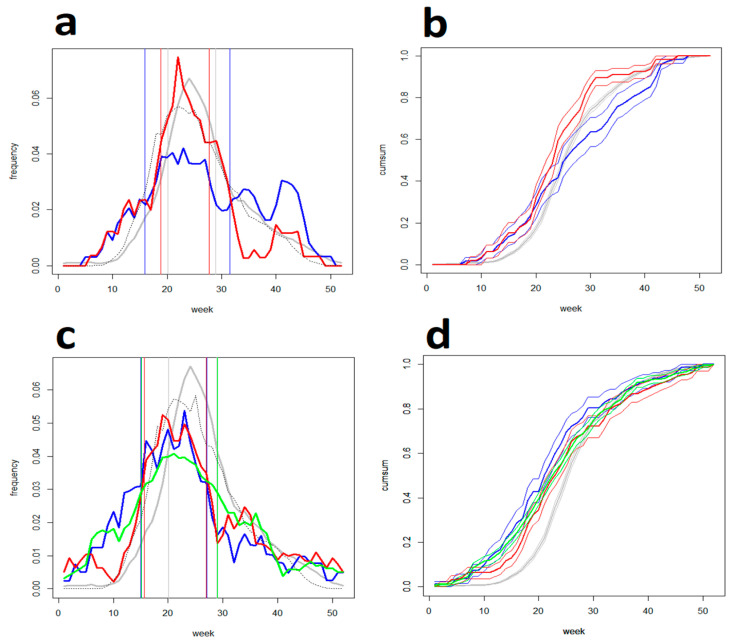
(**a**) Frequency- and (**b**) cumulative diagrams of preventative searches in the Czech Republic: seasonal patterns for the keywords (blue) “tick activity” and (red)” tick occurrence” are collated with (gray) actual tick-bite distribution (Swiss sentinel data) and (dashed) TAI; vertical lines delimit corresponding SPs. (**c**,**d**) Those in Germany: (blue) ”tick weather”, (red) “tick occurrence”, and (green) ”tick risk”. Note that in both countries, (1) the advent of preventative searching precedes the emergence of the risk itself by at least a month; (2) during the spring months of March, April, and May, the volume of preventative searches increases along with the frequency of tick bites; (3) this occurs until the trend’s reversal early in June, ca., 2–4 weeks before tick-bites reach their maximum (the pattern is more clearly pronounced in Germany).

People made tick bite-preventive searches infrequently; nevertheless, preventive searching clearly distinguished the two countries where the system has been implemented from the two control countries (χ^2^: *p* < 0.01) in which these keywords were either absent from the search volumes completely or occurred at a very low frequency (≤8.5% non-zero weeks). At the same time, the prevalence of the country-specific keywords (corresponding with the respective website titles) distinguished the Czech Republic and Germany from each other (Sison–Glaz simultaneous confidence intervals for the keywords “tick activity” and “tick weather” were 0.16–0.27/0–0.05 and 0.1–0.22/0.2–0.65, respectively). This documents that the data reflected intentional preventive searches rather than being generated by chance only (Table 1). Figure 1 illustrates how the preventive searches were distributed over an average year. Both in the Czech Republic and Germany, there were only minor differences from a common pattern seen in individual keywords (detectable mostly in the autumn season). On average, preventive searching in Germany culminated approximately 2 weeks before searching in the Czech Republic notwithstanding comparable TAI distributions. A marked and statistically demonstrable feature—common to both countries—is that tick-bite-preventive searches arose early in winter-spring, far ahead of factual tick-bite risk emergence (contrasting with both TAI and epidemiological tick-bite data).

**Figure 2:**
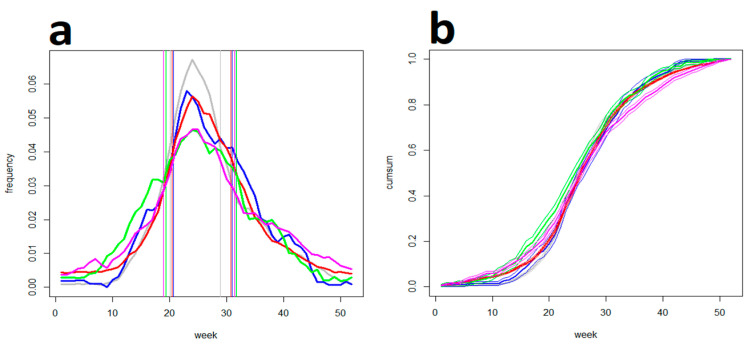
Searches for the keyword “tick bite” (exemplified by ”zeckenstich“ in the German-speaking countries). Shown are (**a**) frequency- and (**b**) cumulative diagrams for (blue) the Czech Republic, (red) Germany, (green) Austria, and (magenta) Switzerland collated with (gray) Swiss sentinel data. Vertical lines delimit corresponding SPs. Note that the distributions are closely related and all peak around the 24th week (mid-June).

**Figure 3:**
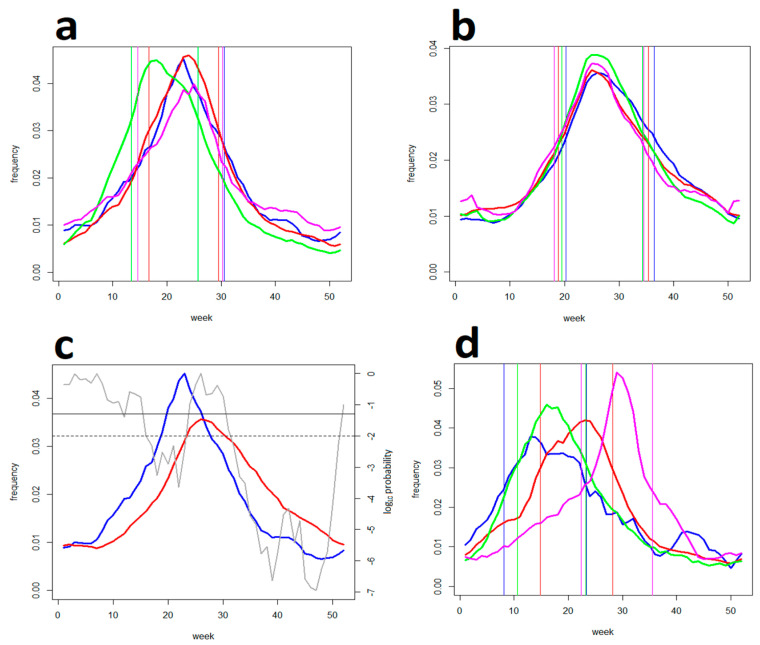
Searches for the keywords (**a**) ”tick-borne encephalitis” and (**b**) “Lyme borreliosis” in (blue) the Czech Rep., (red) Germany, (green) Austria, and (magenta) Switzerland. Vertical lines delimit corresponding SPs. Note that TBE patterns vary somewhat and searches in Austria are advanced, ca., 3–4 weeks before the other countries, whereas LB searches are distributed almost identically in all the four countries. (**c**) Comparison of (blue) TBE and (red) LB patterns exemplified by the Czech data: (grey) the Wilcoxon statistic, (solid) 95%, and (dashed) 99% significance levels (right-hand scale) show the distinctions. Note that the LB searches are significantly delayed (by, ca., 4–5 weeks) after the TBE searches. (**d**) Internet searches for “TBE vaccination” in (blue) the Czech Rep., (red) Germany, (green) Austria, and (magenta) Switzerland. Note that the distributions are country-specific.

Of the post-exposure searches, the keyword “tick bite” exhibited an almost undistinguishable distribution in all the four countries, and closely copied the distribution of actual tick bites reported to the Swiss sentinel system (Figure 2). This is suggestive of a high degree of synchrony both of the tick-bite risk and the population exposure to it over central Europe. In spite of this obvious homogeneity, people in Austria searched for “tick-borne encephalitis” significantly (ca., 1 month) earlier in spring compared to the other three countries where the searches were distributed alike (Figure 3a). In this context, GT data on “TBE vaccination” were also analysed, revealing distinct, country-specific patterns possibly connected with differently broadcasted national vaccination campaigns (Figure 3b), potentially enlightening the Austrian distinctness. In contrast, searches for “Lyme borreliosis” (Figure 3c) were distributed identically in all the four countries, and delayed, ca., 4–5 weeks after the searches for “tick-borne encephalitis”, as documented in Figure 3d.

**Figure 4:**
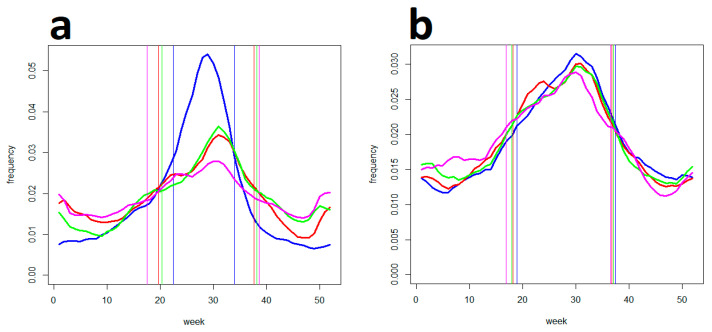
(**a**) Internet searches for four representative resorts/tourist attractions in (blue) the Czech Rep., (red) Germany, (green) Austria, and (magenta) Switzerland. Vertical lines delimit corresponding SPs. Note that in all the four countries, these recreational-activity-associated searches culminate between July and August (around the 30th week). (**b**) “Weather” searches as a proxy of outdoor activities in (blue) the Czech Rep., (red) Germany, (green) Austria, and (magenta) Switzerland. Note that there is no obvious distinction between the diagrams (except for Whitsun holidays), and all peak in July–August consistently with the resort/attraction searches.

Overall, Table 2 puts the data together and gives an idea of the potential of the two national tick-bite risk forewarning systems to influence TBD’s morbidity. Its interpretation relies on a sound assumption that temporal distributions of tick bites in the Czech Republic and Germany compare to that in Switzerland (c.f., Figure 2) where only such cases were systematically registered. The best correspondence between TBD’s and the tick-bite data in Switzerland is found for lags of 2–3 and 3 weeks in the TBE- and LB-reporting, respectively (Table 2). Given the same delays are present in the Czech and German epidemiological data, it reveals that—except for “tick risk” and TBE in Germany—a majority of TBE cases (ca., 55%) and a great majority of LB cases (ca., 70%) are contracted beyond the momentous period of April–July when the prevention systems get/gain the maximum people’s attention. In turn, by far the greatest part of TBD cases (ca., 68%) consistently coincide with a period of the most concentrated recreational/outdoor activities (May–September) as surrogated by the “weather” searches (Figure 4).

### 3.2. Simulations

**Figure 5:**
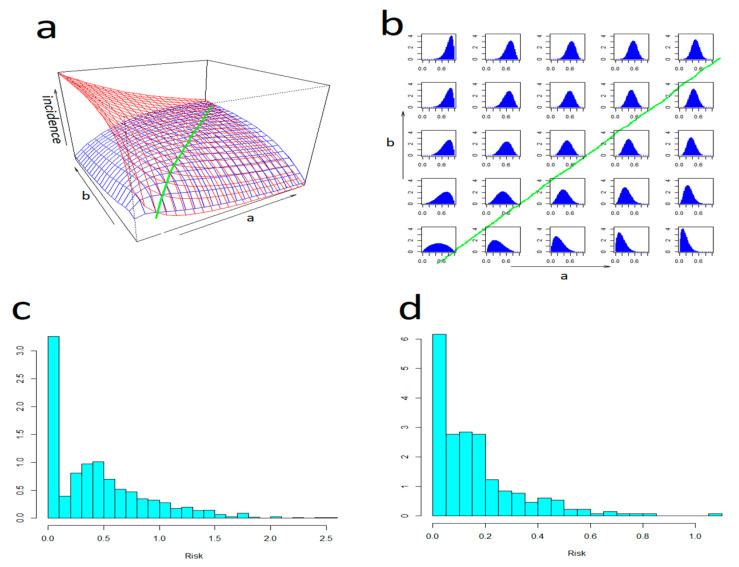
An effect of the distribution of tick-bite risk upon incidence: Panel (**a**) shows simulated incidence for (in blue) exposure kept inversely proportional to tick activity, and (in red) completely random exposure. Panel (**b**) shows base-plane projections of the shape of the risk distribution (each diagram illustrates the shape at the point to which it is centred). Note that the tick-activity-driven incidence is significantly reduced in a range of situations except in a domain—delimited by (in green) an intersection of the two plots—where there is a reversal (typically where the distribution is positive-skewed). Panel (**c**) shows the empirical distribution of the tick-bite risk estimated from a pool of “tick-bite“ searches in the four countries, and panel (**d**) shows that estimated from the tick bite reports to the Swiss sentinel system (”weather“ searches served a population-at-risk denominator). Note that both approaches detected a distinctly positive-skewed distribution (zero-spikes correspond to the winter season of virtually no tick activity and can be ignored).

Figure 5a,b illustrate how the theoretical potential to reduce the incidence by avoiding risk (the higher the risk, the more limited the exposure to it) changes with changing (shape of the) risk distribution. It indicates that—in comparison to spontaneous, haphazard behaviour modelled with random exposure—the warnings-driven exposure has a significant reducing effect in a range of situations. However, on the other hand, it can be counterproductive in a subset of situations when the risk is positive-skew-distributed (i.e., in terms of risk perception when occasions of acceptably low risk outnumber those of cautionary medium and high risk). This leads to incidences in excess of the random model (note that a reversal can indeed occur under real conditions whenever the amount of caution that people use upon being made aware that the risk is low decreases below that they exercised while being uncertain about its actual level). Figure 5c,d show an empirical distribution of the tick-bite risk estimated alternatively from GT- end epidemiological data. It indicates that—in the central European conditions—this distribution is indeed distinctly positive-skewed.

**Figure 6:**
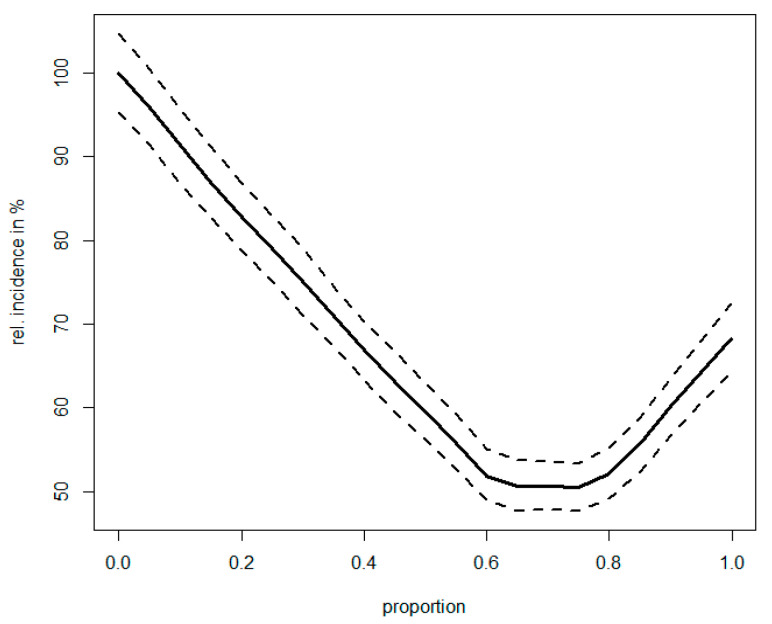
Potential increase in the yield of prevention when allowing for population exposure in addition to tick questing activity. Simulated effect is plotted against the weight that the population exposure is given in the risk assessment, ranging from 0 (solely tick-activity-based prevention) to 1 (solely population-exposure-based prevention). The incidence is expressed relative to a solely tick-activity-based model; solid line shows the mean, and dashed lines indicate 95% confidence interval based on 1000 simulations. Note that in an optimum configuration, theoretical prevention yield is almost twice as high compared to the exclusively tick-activity-based model.

Finally, Figure 6 illustrates a scenario of what would occur with the TBD’s incidence if the warning system weighed jointly the questing activity of ticks as well as human outdoor activity. The simulation shows that there is a capacity for further reduction in the incidence of up to, ca., 50% if both factors are taken into account in a balanced manner, compared to the established design when warning urgency reflects tick activity alone.

## 4. Discussion

Internet forecasts warning against a weather of high tick activity constitute a novel instrument to counter the contemporary increase in TBD incidence experienced in Europe and elsewhere in the mid-latitudes [34]. Broad and instant information about the actual level of risk could dissuade people from coming into contact with ticks and bring about a deceleration in the emergence of new disease cases. The present analysis, however, revealed that the efficiency of existing implementations is not optimum, and that they might even be counterproductive at times. Seemingly, the collected data show that—in both the Czech Republic and Germany—on average, fewer TBD cases were contracted at the ticks’ peak time from April to July (culminating toward the end of May [31,35])—which was the period when people also made the largest volume of preventive searches. However, what appears as resulting from a precaution in risk-aware people cannot be straightforwardly attributed to the Internet warnings, as the preventive searches were too infrequent to have such a profound influence. The circumstance rather seems to be entirely incidental and most plausibly attributable to a preponderance of TBD cases in the main holiday time during the hottest months of July and August (culminating around mid-July [36,37]), past the ticks’ maximum. At that time, the tick-bite warnings may not be as urgent/sustaining vigilance as in the springtime, but recreational activities—and hence human–tick contacts—are at their highest ever level. Since the yield of prevention (measured in terms of the number of cases of tick-bite/infection avoided per season) would in theory be maximal when public vigilance about tick bite prevention is maintained in a counterbalance relation to how likely human–tick encounters are to occur (not proportional to sole abundance of questing ticks, irrespective of human presence), these implementations are inherently suboptimum.

As opposed to a solely weather-driven model of tick-bite risk, disregard for the size of the exposed population is automatically precluded in the alert systems that rely on volunteer-supplied information on tick occurrence (e.g., [38]; “Tick Radar” https://www.tekenradar.nl/ (accessed on 20 July 2020), or mobile applications “TickApp” https://thetickapp.wordpress.com/ (accessed on 20 July 2020), and “Tick” https://www.zhaw.ch/en/lsfm/business-services/institute-of-natural-resource-sciences/ticks/tick-app/ (accessed on 1 January 2019)). In this approach, the degree of tick-bite risk is deduced from the influx rate of volunteers’ reports on chance encounters with a tick, events that are, indeed, distributed proportionally to the density of the exposed population. Thus—given the same tick activity conditions—this system signals a higher degree of risk (i.e., higher frequency of new case emergence) the more people that are exposed, which in principle takes effect against increased human–tick contacts. A disadvantage of this approach is, however, that the information is delayed, subject to volunteer error, and that less frequented areas/periods may be critically under-sampled [39].

In order to take advantage of the forewarning potential of the weather-driven system—and jointly to offset the deficiency in the population exposure regard—the algorithm for assessing the risk severity should be modified. In effect, the prevention yield could hypothetically be improved to a level almost twice as high as that gained without taking the population exposure into account (contingent on what weight this factor is given in the calculations of risk rating, of course). If nothing else, a certain increase in the yield would pre-compensate for situations where the alert system is liable to do a potential disservice to prevention (i.e., when the alert-regulated case rate overtakes the spontaneous rate). To reach this goal, a realistic appraisal of the amount of people exposed outdoors to tick bites is needed. Epidemiological modelling of TBDs relies on surrogates: it employs, for example, the fact that human outdoor/recreational activities are correlated with some environmental factors such as sunshine duration [40]; however, physical variables do not explain the population exposure completely enough for the given purpose. One alternative approach utilizes the fact that the amount of overnight accommodation booked by tourists (especially in rural destinations) closely follows the pattern of recreational/touristic activities [41]. Unfortunately, these data (reported out of duty by lodging providers to local authorities) are not ready for operative usage. Nevertheless, it was demonstrated that the mere volume of Internet queries about tourist destinations can fulfil the same surrogate function, and of particular use is that it can be traced in almost real-time, compared to the overnight accommodation data [42]. Eventually, as people plan recreational activities in conformity with weather conditions (e.g., [43]), it is not surprising that Google searches for the keyword “weather” have proved themselves in a collation (on Figure 4) to closely parallel the tourist destination searches. The circumstance that any weather-driven tick-bite warning system hosted on a meteorological webpage could make use of the page’s access statistic (giving a timely estimate of people’s actual concern over outdoor conditions) makes the “weather” searches a candidate for a true real-time surrogate for the population’s exposure outdoors.

Naturally, a prerequisite for the desired effect of prevention is that the underlying predictions (both of tick-questing activity itself and the weathercast behind it) are realistic enough. Their accuracy cannot be, however, 100% trusted, and it was beyond the capacity of this study to perform their validation. Nevertheless, it can be presumed that—although too many departures from reality could diminish or even nullify the prevention yield—possible inaccuracies are unlikely to completely upset the purpose of tick-bite mitigation once the condition of an overall neutral prediction error is maintained (as much underestimation of the risk as overestimation). Various approaches have been proposed as to how to predict the degree of activity of *I. ricinus* under certain weather conditions. Apart from an authentic yet impractical direct monitoring of questing activity on special phenological plots [44], the methodology relies on machine learning, weather data, and algorithms ranging from a simple cross-tabulation of empirical activity indices against the two most decisive environmental variables—ambient temperature and humidity—to much more sophisticated multivariate statistical models that employ additional contributing variables and allow for their accumulated influence over time, e.g., [45,46,47,48,49,50,51]. Objective comparison of these methods is, however, hindered by differences in measuring prediction success.

In summary, both countries where the prevention system in question is established overlap with a highly endemic zone of TBDs in central Europe and, in recent years, have experienced the two highest numbers of TBE cases in the EU [34]. To address the epidemiological challenge, any measure of tick bite prevention—however sporadically adopted in the target populations—can be an asset. Similarly, any correction in the existing implementation of a measure that leads to better performance is desired. This study argues that a simple avenue to increase the prevention yield of this system is to apply the joint probability of human–tick contacts rather than solely tick-questing activity as a gauge for scaling the hazard. As a surrogate for the factor of human presence, it proposes using the visit traffic of a (weather) website hosting the system. This would furnish the system with a feedback—similar to the volunteer tick-monitoring approach—between the access frequency to an information source relevant to outdoor activities—plausibly tracking the proportion of the population actually exposed outdoors and being at risk—and the urgency of tick-bite warnings, maintaining the appropriate level of public vigilance regarding tick bite prevention even beyond tick maxima. More studies will be needed to further optimize this system with respect to collective prevention.

## Figures and Tables

**Table 1:** Google-Trends search volumes per country and keyword: queries are classified (# in column 1) as (1) preventive searches, (2) post-exposure searches, and (3) tourism/recreation-associated searches. National equivalents of the monitored keywords/alternatives are shown in quotation marks—German keywords were common to Germany, Austria, as well as Switzerland where it is used by, ca., 63% of population (French and Italian keywords were omitted due to their scarcity). Values in brackets show scores for each keyword’s search frequency (sum of relative search volumes is given over % of non-zero weeks; zeroes indicate no searches). Note the contrast in interests in preventive information on tick-bite risk (e.g., “tick-activity”, “tick-weather”) between countries where forecasts are provided and those where they are not.

#	Search Query	Czech Rep.	Germany	Austria	Switzerland
1	tick activity	„aktivita klíšťat“ (2285/20.8)	„zeckenaktivität“ (2251/15.8)	(0)	(0)
	tick occurrence	„výskyt klíšťat“ (2085/20.8)	„zecken vorkommen“ (2804/30.0)	(0)	(0)
	tick weather	(0)	„zeckenwetter“ (3141/26.2)	ditto * (193/0.1)	ditto * (178/0.1)
	tick risk	(0)	„zeckengefahr“ (6037/55.8)	ditto * (1596/7.3)	ditto * (1158/8.5)
2	tick bite	„kousnutí klíštětem“ (3492/45.8)	„zeckenstich/-biss“ (6417/97.3, 5724/100)	ditto * (5875/52.3, 5236/99.2)	ditto * (5452/75.8, 5294/98.8)
	tick-borne encephalitis	„klíšťová encefalitida“ (5087/96.5)	„FSME“ (7066/100)	ditto * (6287/100)	ditto * (5023/100)
	Lyme borreliosis	„Lymská borelióza“ (10,090/100)	„borreliose“ (10,499/100)	ditto * (8159/100)	ditto * (6018/100)
3	weather	„počasí“ (9745/100)	„wetter“ (10,464/100)	ditto * (11,665/100)	ditto * (13,603/100)
	resort/tourist attraction	Lipno dam (4681/100)	Königssee (9515/100)	Hallstatt (8081/100)	Interlaken (12,472/100)

*: common German keywords were applied.

**Table 2:** Proportions (in %) of reported tick-borne disease cases that fall within SP of Internet searches (specified in the 2nd column) given the reporting lags indicated (in the 3rd row); “weather” searches are shown for comparison. The Swiss data serve for lag-calibration: the maxima of coincidence (marked in bold) between tick bite ”events” (localized by the date of medical consultation or Internet search) and reported cases indicate that, on average, TBE- and LB reports are, ca., 2–3 and 3 weeks lagging after actual dates of infection, respectively.

	Search Query	Tick-Borne Encephalitis					Lyme Borreliosis				
		Lag in Weeks					Lag in Weeks				
		0	1	2	3	4	0	1	2	3	4
Czech Rep.	tick activity	28.9	34.3	39.8	45.2	50.2	25.0	27.7	30.6	33.8	37.0
	tick occurrence	32.5	37.0	41.1	44.6	47.2	20.9	22.8	24.7	26.5	31.2
	weather	68.5	70.2	71.7	73.1	74.4	54.2	56.1	57.7	59.1	60.1
Germany	tick weather	31.4	38.0	44.2	49.6	54.1	23.9	28.2	32.5	36.7	40.6
	tick occurrence	31.2	37.6	43.5	48.6	52.2	23.4	27.6	31.7	36.0	39.4
	tick risk	44.8	50.7	55.8	60.2	63.5	33.6	38.1	42.1	45.9	49.4
	weather	75.8	76.7	76.9	76.6	76.2	65.1	67.6	69.5	70.7	70.9
Switzerland	tickbite (-stich)	58.3	60.9	61.8	**63.2**	61.6	54.3	55.4	56.7	**57.4**	56.2
	tick bite (-biss)	52.8	56.6	58.7	**60.3**	59.1	51.0	51.9	52.7	**54.3**	53.5
	*sentinel data*	51.1	54.4	**57.2**	56.3	55.8	48.6	49.2	50.3	**50.4**	48.8

## Data Availability

The datasets analyzed in this study are freely available from public sources referred to in the text.

## Supplementary Materials

The following supporting information can be downloaded at https://www.mdpi.com/article/10.3390/life13091908/s1, Supplementary File S1: A concept of “summit period”.
