# Supplementary material for: Tick-Bite “Meteo”-Prevention: An Evaluation of Public Responsiveness to Tick Activity Forecasts Available Online
Source: Life (Basel). 2023 Sep 14;13(9):1908. doi: 10.3390/life13091908 (PMC10533051; doi:10.3390/life13091908)
Supplement: Supplementary file 1 [file life-13-01908-s001.zip › life-2559828-supplementary.pdf]

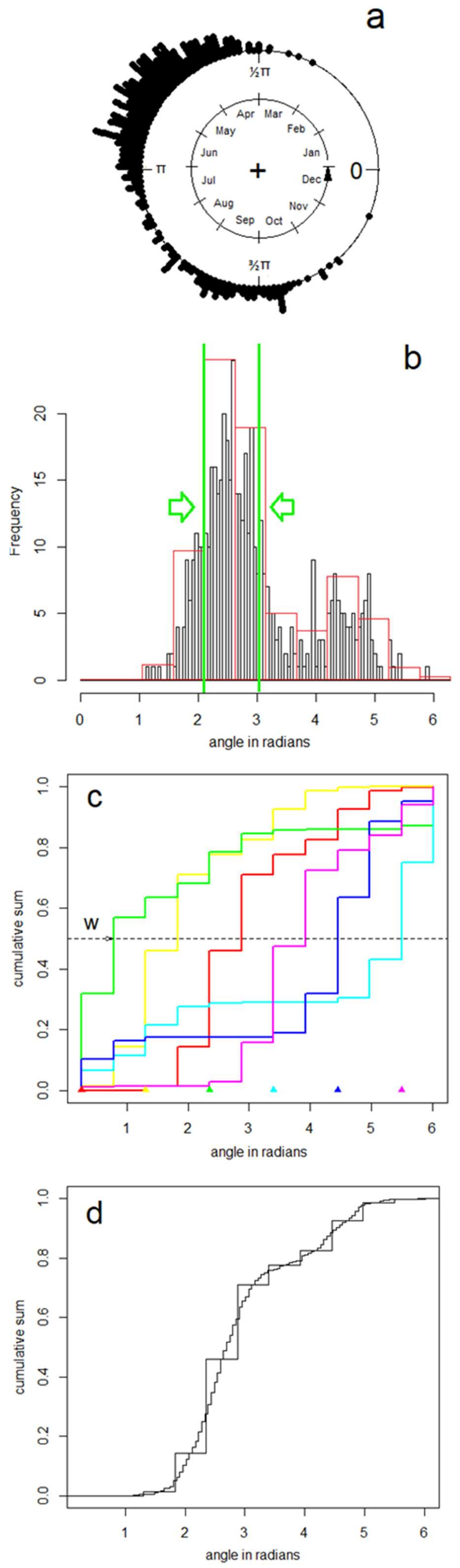

This material explains the concept of “summit period” (SP), and how it has been applied in the present analysis. Tick bites, disease cases, Internet searches, etc. are thereafter termed ‘events’, and SP denotes that time period in the year in which the denser half of events are registered. The logic behind focusing on the denser half of the data is to suppress the influence of outlying off-seasonal observations and potential artefacts upon delimitation of the main season of tick activity.

How can a „summit period“ be identified ? The first step is “wrapping” data around the circumference of a 365-day circle to map events onto a circular (radian) scale (because the problem is better addressed circularly than sequentially). Figure (a) illustrates synthetic data (a mixture of two von Mises, i.e. ‘circular normal’ distributions) that imitates bimodal seasonal activity of the “common tick”. In Figure (b), the same data is plotted as a more conventional histogram on a (0, 2π) scale – for better clarity, computations are illustrated on a rough, ‘monthly’-aggregated data outlined in red. The basic algorithm is familiar: (1) sorted data is divided into *n* bins, (2) *n* cumulative distributions are calculated in the direction of the time arrow in Fig. a, each time shifting the starting point of cumulation by the width of one bin ahead (that is, in the given example, diagrams are calculated for January-December, February-January, …, and December-November, respectively), and (3) the one that exhibits the steepest ascent next to the origin (i.e. arrives first at the 50% level) indicates the SP. Figure (c) shows a collation of six selected graphs (every second graph was omitted for clarity) translated to zero for better comparison, while marks on the horizontal axis indicate where they in fact originate. The green line clearly conforms to the specification, and the interval between points to where it starts (the green mark on X) and where it reaches 50% (the green mark shifted by “w”) covers the denser half of the data; it is projected onto Figure (b) in green. Using finer binning and/or interpolation, its boundaries can be estimated with ‘arbitrary’ precision (cf. Figure d). With the exception of some marginal conditions, SP is continuous and unique, and doesn’t necessarily include extremes (possible outliers!).

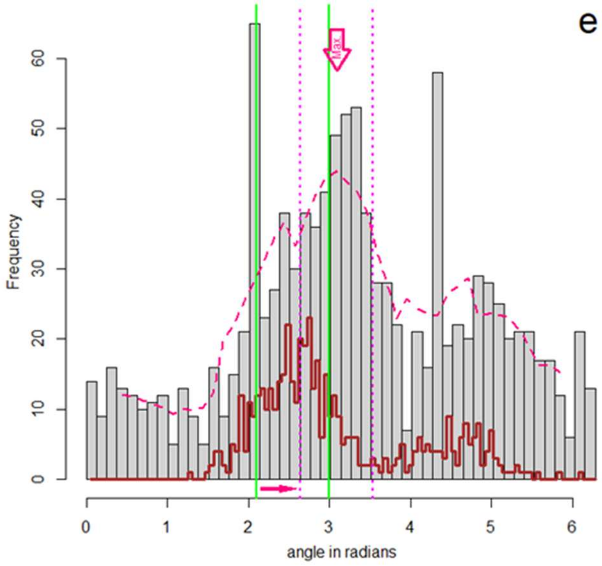

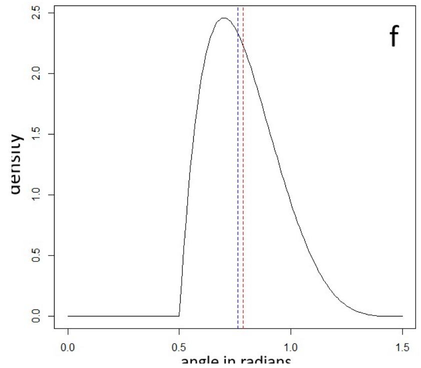

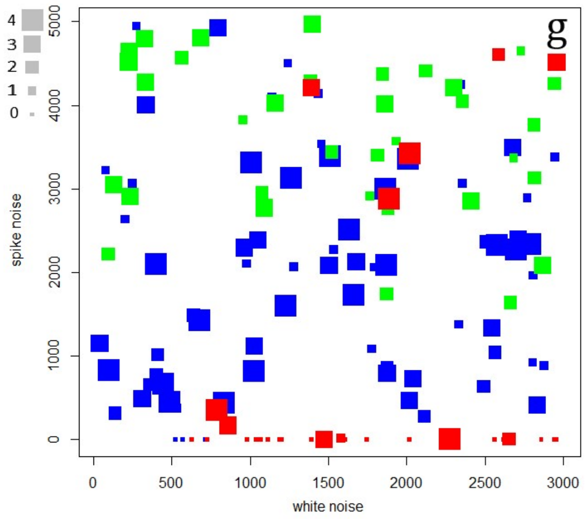

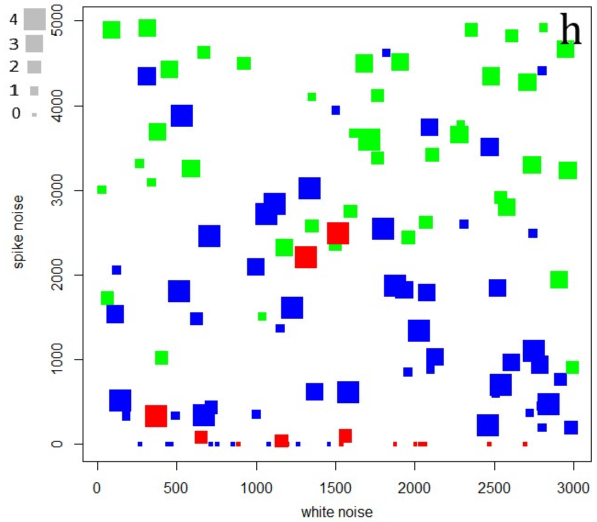

Although SP applies rather to descriptive analysis, maximum overlap of SP regions outperformed some established statistics when applied as a criterion in estimating lags between data time series on causal events (typically tick bites) and effect events (such as post-exposure searches or disease reporting). The idea is simple as illustrated in Figure (e): the brown diagram represents causal events (the same as in Fig b), and the grey histogram shows them shifted by a randomly generated lag (Fig, f; beta-distributed) and mixed with "noise". Two random admixtures were simulated: uniformly distributed "white noise", and "spike noise" – imitating intermittent surges in Internet searching stirred up by media news (triangularly-distributed). The computation is simple: (1) data on the causal events yields SP (in green), (2) that is then shifted bin by bin as a running window ahead in time, (3) until the sum of effect events that fall inside it (violet dashed line) is maximized (violet arrow), and (4) the separation between the initial and shifted SP's positions approximates the lag. Note, that nothing more than an Epi Info database is needed for sorting out cases falling between two calendar dates (cf. Table 2).

Figures (g, h) document a 'validation' experiment conducted to compare performance of the SP-approach with two alternative approaches in use for matching seasonal patterns, namely: difference in means (DM), and lagged correlation (LC). Altogether 250 combinations of time delay (2 weeks – 2 months), white noise (0-3000 events), and spike noise (0-5000 in 0-4 clusters) were simulated, each replicated 100x, and mean squared differences evaluated. Figures (g and h) show results for delays < 5 weeks, and >5 weeks respectively. The respective blue, red, and green marks show which of DM, LC, and SP outperformed the others, while size of the marks indicate the number of spikes (grey). As could be expected SP had the best scores at higher noise levels.
